# Italian guidelines for management and treatment of hyperbilirubinaemia of newborn infants ≥ 35 weeks’ gestational age

**DOI:** 10.1186/1824-7288-40-11

**Published:** 2014-01-31

**Authors:** Costantino Romagnoli, Giovanni Barone, Simone Pratesi, Francesco Raimondi, Letizia Capasso, Enrico Zecca, Carlo Dani

**Affiliations:** 1 Division of Neonatology, Department of Pediatrics, Catholic University S H, Largo A. Gemelli, 8, Rome 00168, Italy; 2 Section of Neonatology, Department of Surgical and Medical Critical Care, Careggi University, Hospital of Florence, Florence, Italy; 3 Department of Pediatrics, Federico II University of Naples, Corso Umberto I, 40, Napoli 80138, Italy

**Keywords:** Hyperbilirubinaemia, Jaundice, Newborn, Discharge, Guidelines

## Abstract

Hyperbilirubinaemia is one of the most frequent problems in otherwise healthy newborn infants. Early discharge of the healthy newborn infants, particularly those in whom breastfeeding is not fully established, may be associated with delayed diagnosis of significant hyperbilirubinaemia that has the potential for causing severe neurological impairments. We present the shared Italian guidelines for management and treatment of jaundice established by the Task Force on hyperbilirubinaemia of the Italian Society of Neonatology.

The overall aim of the present guidelines is to provide an useful tool for neonatologists and family paediatricians for managing hyperbilirubinaemia.

## Background

Hyperbilirubinaemia is a very common condition. The prevention, detection and management of jaundice remains a challenge especially because of early discharge of healthy late preterm and full term newborn infants [1-3]. Early discharge of the healthy newborn, particularly those in whom breastfeeding is not fully established, may be associated with delayed diagnosis of severe hyperbilirubinaemia [1,4,5]. A recent survey performed by Dani et al. [6] showed that the management of hyperbilirubinaemia is not homogeneous in Italy. Therefore the Task Force on hyperbilirubinaemia of the Italian Society of Neonatology drew up national guidelines for management of jaundice in the newborn and established a national registry of kernicterus and hyperbilirubinaemia in order to monitor the incidence of cases of kernicterus and severe hyperbilirubinaemia in Italy over time.

### Focus of guidelines

The overall aim of this guidelines is to provide an useful tool for neonatologists and family paediatricians for managing hyperbilirubinaemia. These recommendations promote an approach based on the importance of universal systematic assessment for the risk of severe hyperbilirubinaemia, close follow-up, and prompt intervention when indicated. The statement fully applies to the care of infants at 35 or more weeks of gestation, while only some recommendations could be used for newborn infants with lower gestational age (GA).

### Methods of statement development

Literature searches were last updated in September 2013. Guidelines from AAP [7-9], NICE [10,11], Canadian Pediatric Association [12], Nederland Neonatal Research Network [13], Norwegian Pediatric Society [14], Israel [15], New Zealand [16], Australia (Queensland) [17], India [18], Spain [19], Switzerland [20] were considered. Studies conducted in the neonatal units of Lazio were also evaluated to obtain recommendations more suitable for Italian population [21-23].

The hierarchy of evidence from the Centre for Evidence-Based-Medicine was applied (see Table 1) [24].

**Table 1:** The hierarchy of evidence from the centre for evidence-based-medicine

**Level**	**Definition**
1a	Systematic review (with homogeneity) of RCTs
1b	Individual RCT (with narrow confidence interval)
2a	Systematic review (with homogeneity) of cohort studies
2b	Individual cohort study
3a	Systematic review (with homogeneity) of case–control studies
3b	Individual case–control study
4	Case-series (and poor quality cohort and case–control studies)
5	Expert opinion without explicit critical appraisal, or based on physiology, bench research or “first principles”

### Acute bilirubin encephalopathy

The main aim of these guidelines is to avoid cases of acute bilirubin encephalopathy (ABE) by establishing levels of serum bilirubin at risk of neurologic damage. A total serum bilirubin (TSB) of 20 mg/dl should not be exceeded in the first 96 hours of life in term newborns. After 96 hours of life a TSB of 25 mg/dl should be avoided (evidence level 2b), since the efficacy of Blood–brain-Barrier increases with postnatal age. The safe bilirubin level is lower for preterm infants: 12 mg/dl for babies with GA ≤ 30 weeks and 15 mg/dL for babies with GA 31–36 weeks [25,26].

Apart from TSB level, gestational age and postnatal age, some more risk factors for ABE should also be considered:

• Birth asphyxia

• Severe hypothermia (Tc < 36°C for > 6 hours)

• Respiratory failure (RDS, pneumonia, meconium aspiration syndrome)

• Prolonged acidosis (pH < 7.20 for > 6 hours)

• Severe hypoglycaemia (glucose < 45 mg/dl for > 12 hours)

• Severe haemolysis

• Sepsis and meningitis

• Drugs impairing bilirubin/albumin binding

### Bilirubin measurement

All infants should be routinely monitored for the development of jaundice. The ability of physicians and other health care providers to recognise clinically significant jaundice and predict bilirubin levels based on the coephalo-caudal progression of jaundice is limited (evidence level 1b) [27-31].

Therefore each jaundiced newborn should receive a bilirubin measurement. Transcutaneous bilirubin (TcB) can be used as first step in order to reduce the number of invasive and painful blood sampling. BiliCheck TM (Respironics, Marietta, GA – USA) and JM-103 (Drager Medical Inc, Telford, Pennsylvania) are the two most used bilirubinometers in the studies conducted in the Italian population and both had good correlation with TSB values [21,22,32,33]. TSB measurement is always necessary when the level of bilirubin is high and for therapeutic decisions (evidence level 1b).

### Prediction of hyperbilirubinaemia

**Figure 1:**
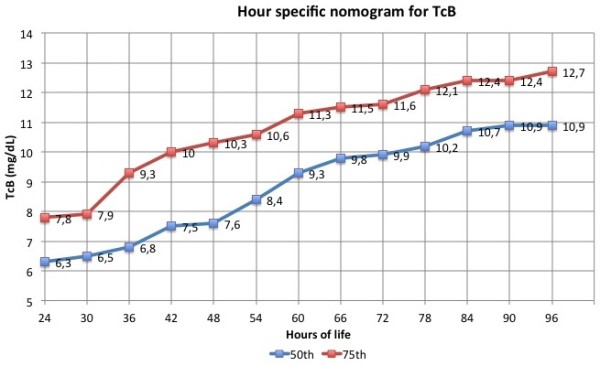
Depicts hour-specific percentile based nomograms for TcB.

**Figure 2:**
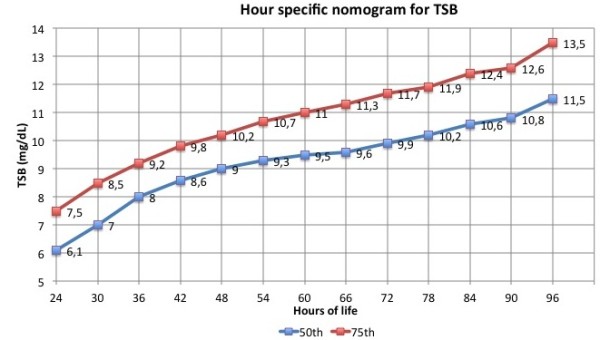
Depicts hour-specific percentile based nomograms for TSB.

The evaluation of jaundice is now facilitated by the availability of different nomograms for both serum [34] and transcutaneous bilirubin [21,35,36]. However, a nomogram could not perform well in one specific setting if the racial, genetic and environmental background of that setting are too different from that of the reference population. Furthermore, predictive tools should be developed in one sample and validated in another one. Hour-specific percentile based nomograms have been developed in cohorts of healthy Italian full term neonates or Italian neonates with ≥ 35 weeks’ gestational age using serial measurements of TcB and TSB (Tables 2 and 3; Figures 1 and 2) [21,23]. In a second phase the predictive ability of these nomograms has been evaluated. Sensitivity, specificity, positive and negative predictive value of percentiles in predicting significant hyperbilirubinaemia (defined as TSB > 17 mg/dL or need for phototherapy) are listed in Tables 4 and 5.

**Table 2:** Values of TcB corresponding at the 50th and 75th percentile of the hour-specific nomogram

**h**	**50th**	**75th**	**h**	**50th**	**75th**	**h**	**50th**	**75th**
**24**	**6, 3**	**7, 8**	49	7, 7	10, 4	73	10	11, 7
25	6, 3	7, 8	50	7, 8	10, 4	74	10	11, 8
26	6, 4	7, 8	51	8	10, 5	75	10, 1	11, 9
27	6, 4	7, 9	52	8, 1	10, 5	76	10, 1	11, 9
28	6, 4	7, 9	53	8, 3	10, 6	77	10, 2	12
29	6, 5	7, 9	**54**	**8, 4**	**10, 6**	**78**	**10, 2**	**12, 1**
**30**	**6, 5**	**7, 9**	55	8, 6	10, 7	79	10, 3	12, 2
31	6, 6	8, 1	56	8, 7	10, 8	80	10, 4	12, 2
32	6, 6	8, 4	57	8, 9	11	81	10, 5	12, 3
33	6, 7	8, 6	58	9	11, 1	82	10, 5	12, 3
34	6, 7	8, 8	59	9, 2	11, 2	83	10, 6	12, 4
35	6, 8	9, 1	**60**	**9, 3**	**11, 3**	**84**	**10, 7**	**12, 4**
**36**	**6, 8**	**9, 3**	61	9, 4	11, 3	85	10, 7	12, 4
37	6, 9	9, 4	62	9, 5	11, 4	86	10, 8	12, 4
38	7, 1	9, 5	63	9, 6	11, 4	87	10, 8	12, 4
39	7, 2	9, 7	64	9, 6	11, 4	88	10, 8	12, 4
40	7, 3	9, 8	65	9, 7	11, 5	89	10, 9	12, 4
41	7, 5	9, 9	**66**	**9, 8**	**11, 5**	**90**	**10, 9**	**12, 4**
**42**	**7, 5**	**10**	67	9, 8	11, 5	91	10, 9	12, 5
43	7, 5	10, 1	68	9, 8	11, 5	92	10, 9	12, 5
44	7, 6	10, 1	69	9, 9	11, 6	93	10, 9	12, 6
45	7, 6	10, 2	70	9, 9	11, 6	94	10, 9	12, 6
46	7, 6	10, 2	71	9, 9	11, 6	95	10, 9	12, 7
47	7, 6	10, 3	**72**	**9, 9**	**11, 6**	**96**	**10, 9**	**12, 7**
**48**	**7, 6**	**10, 3**						

**Table 3:** Values of TSB corresponding at the 50th, 75th and 90th percentile of the hour-specific nomogram

**h**	**50th**	**75th**	**90th**	**h**	**50th**	**75th**	**90th**	**h**	**50th**	**75th**	**90th**
**24**	**6.1**	**7.5**	**8.9**	49	9.0	10.3	11.9	73	10.0	11.7	13.2
25	6.2	7.7	9.1	50	9.1	10.4	12.0	74	10.0	11.8	13.3
26	6.4	7.8	9.2	51	9.1	10.4	12.1	75	10.1	11.8	13.3
27	6.5	8.0	9.3	52	9.2	10.5	12.2	76	10.1	11.8	13.4
28	6.7	8.2	9.5	53	9.2	10.6	12.3	77	10.2	11.9	13.4
29	6.8	8.3	9.6	**54**	**9.3**	**10.7**	**12.4**	**78**	**10.2**	**11.9**	**13.5**
**30**	**7.0**	**8.5**	**9.7**	55	9.3	10.8	12.5	79	10.3	12.0	13.5
31	7.2	8.6	9.9	56	9.3	10.8	12.5	80	10.3	12.1	13.6
32	7.3	8.7	10.1	57	9.3	10.9	12.6	81	10.4	12.1	13.7
33	7.5	8.9	10.2	58	9.4	10.9	12.7	82	10.5	12.2	13.7
34	7.7	9.0	10.5	59	9.4	11.0	12.8	83	10.6	12.3	13.8
35	7.9	9.1	10.6	**60**	**9.5**	**11.0**	**12.9**	**84**	**10.6**	**12.4**	**13.8**
**36**	**8.0**	**9.2**	**10.8**	61	9.5	11.1	12.9	85	10.6	12.4	13.9
37	8.1	9.3	10.8	62	9.5	11.1	12.9	86	10.7	12.5	14.0
38	8.2	9.4	10.9	63	9.5	11.2	12.9	87	10.7	12.5	14.1
39	8.3	9.5	10.9	64	9.6	11.2	13.0	88	10.7	12.5	14.2
40	8.4	9.6	11.0	65	9.6	11.3	13.0	89	10.8	12.6	14.3
41	8.5	9.7	11.1	**66**	**9.6**	**11.3**	**13.0**	**90**	**10.8**	**12.6**	**14.4**
**42**	**8.6**	**9.8**	**11.1**	67	9.6	11.4	13.0	91	10.9	12.7	14.5
43	8.7	9.9	11.2	68	9.6	11.4	13.1	92	11.0	12.9	14.6
44	8.7	9.9	11.3	69	9.7	11.5	13.1	93	11.2	13.0	14.7
45	8.8	10.0	11.5	70	9.8	11.6	13.1	94	11.3	13.2	14.8
46	8.9	10.1	11.6	71	9.8	11.7	13.2	95	11.4	13.4	14.9
47	8.9	10.2	11.7	**72**	**9.9**	**11.7**	**13.2**	**96**	**11.5**	**13.5**	**15.0**
**48**	**9.0**	**10.2**	**11.8**								

**Table 4:** Ability of TcB measurements over the 50th, the 75th and the 90th percentile of TcB nomogram to predict significant hyperbilirubinaemia, for designated time periods

**Hours of life**	**TP**	**FN**	**TN**	**FP**	**Sensitivity**	**Specificity**	**PPV**	**NPV**
**24 - 48 hours**								
<50th percent	27	0	161	460	100%	25.9%	5.5%	100%
<75th percent	25	2	429	192	92.6%	69.1%	11.5%	99.5%
<90th percent	14	13	564	57	51.9%	90.8%	19.7%	97.7%
**49 - 72 hours**								
<50th percent	21	0	410	615	100%	40%	3.3%	100%
<75th percent	21	0	702	323	100%	68.5%	6.1%	100%
<90th percent	18	3	888	137	85.7%	86.6	11.6%	99.7%
**73 - 96 hours**								
<50th percent	7	0	246	220	100%	52.8%	3.1%	100%
<75th percent	7	0	348	118	100%	74.7%	5.6%	100%
<90th percent	5	2	409	57	71.4%	87.8%	8.1%	99.5%

**Table 5:** Ability of TSB measurements over the 50th, the 75th and the 90th percentile of TSB nomogram to predict significant hyperbilirubinaemia, for designated time periods

**Hours of life**	**TP**	**FN**	**TN**	**FP**	**Sensitivity**	**Specificity**	**PPV**	**NPV**
**24 - 48 hours**								
<50th percent	24	1	357	263	96.0%	57.6%	8.4%	99.7%
<75th percent	22	3	479	141	88.0%	77.3%	13.5%	99.4%
<90th percent	12	13	576	44	48.0%	92.9%	21.4%	97.8%
**49 - 72 hours**								
<50th percent	23	0	657	368	100%	64.1%	5.9%	100%
<75th percent	22	1	860	165	95.7%	83.9%	11.8%	99.9%
<90th percent	17	6	975	50	73.9%	95.1%	25.4%	99.4%
**73 - 96 hours**								
<50th percent	7	0	322	145	100%	69.0%	4.6%	100%
<75th percent	7	0	409	58	100%	87.6%	10.8%	100%
<90th percent	6	1	446	21	85.7%	95.5%	22.2%	99.8%

On the base of these studies the Task Force on hyperbilirubinaemia of the Italian Society of Neonatology drew up the following recommendations for infants with GA ≥ 35 weeks (evidence level 1b).

***Recommendations***:

• All jaundiced newborn infants should be tested with a TcB measurement and the value should be plotted on the hour-specific nomogram for TcB measurements (Table 2).

• If the TcB measurement is > 75th percentile, a serum determination of bilirubin should be performed and the value should be plotted on the nomogram for TSB measurements (Table 3).

• Newborn infants with a TSB value < 50th percentile in the first 48 hours of live and babies with a value < 75th percentile after 48 hours of life are not at risk of hyperbilirubinaemia and do not require further evaluations.

• Newborn infants with a TSB value > 50th percentile in the first 48 hours of live and babies with a value > 75th percentile after 48 hours of life are at risk of hyperbilirubinaemia and should be tested again after 24 or 48 hours according to the hours of life and the presence of clinical risk factors.

At present, there are no nomograms able to predict hyperbilirubinaemia in infants with GA < 35 weeks. Therefore bilirubin measurements should be decided according to GA and treatment thresholds (evidence level 5).

### Treatment: phototherapy

There is no reliable evidence to inform the choice of thresholds for starting phototherapy. For this reasons guidelines report different recommendations. In determining the bilirubin thresholds for treatment, the Italian Society of Neonatology considered as primary aim to choose a threshold allowing a large margin of safety, being the threshold for starting phototherapy less than that for performing exchange transfusion, and not so low to become unnecessary.

**Figure 3:**
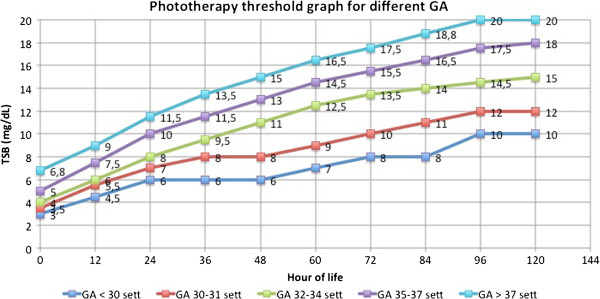
**The graph shows the thresholds for phototherapy.** Total bilirubin was plotted against age in hours. The groups of GA were represented with different lines.

The thresholds for phototherapy are showed in a graph in which total bilirubin is plotted against age in hours. The groups of GA (GA < 30; 30–31; 32–34; 35–37; > 37) are represented by different lines (Figure 3).

***Recommendations*:**

• Phototherapy should be administered for irradiating the most of the infant’s skin.

• Intensive phototherapy with an irradiance > 35 μW/cm^2^/nm is recommended. Daylight, blue light, conventional or blue LED phototherapy can be used (evidence level 1a).

• Fiberoptic phototherapy can be used even if it is less effective than conventional phototherapy and require more prolonged treatment (evidence level 1a) [37].

• Serum bilirubin should be tested 4–8 hours after the beginning of phototherapy or earlier if TSB < 3 mg/dL less than threshold for exchange transfusion. Subsequently, TSB should be assessed every 12–24 hours to monitor the treatment effectiveness.

• In case of treatment failure, multiple phototherapy should be started adding a fiberoptic device to the conventional treatment to increase the skin’s exposure (evidence level 1a) [37].

• Phototherapy should be discontinued once the bilirubin levels are below the threshold value for treatment on two consecutive measurements, 6–12 h apart. This would avoid keeping babies under phototherapy longer than necessary [38] (evidence level 1b).

• Serum bilirubin should be checked 12–24 hours after discontinuation of phototherapy the occurrence of post-phototherapy bilirubin rebound [39] (evidence level 4).

• Infants with either Rhesus or ABO haemolytic disease should be immediately treated with phototherapy once the diagnosis is made.

Hydration status should be monitored during phototherapy by daily weighing of the baby, assessing wept nappies and, if required, monitoring electrolytaemia. Breastfeeding should not be discontinued, because its interruption is associated with an increased frequency of stopping breastfeeding by one month (evidence level 2b) [40]. Italian Society of Neonatology suggests brief suspensions (up to 30 minutes) of phototherapy for allowing breastfeeding.

Breastfed babies should not be routinely supplemented with formula, water or dextrose water for the treatment of jaundice (evidence level 1b) [7,41]. The need for additional fluids during phototherapy should be considered only when the daily weight loss is higher then 5%, or when breast milk is not sufficient to permit the full feeding.

### Exchange transfusion (ET)

Exchange Transfusion is recommended for newborn infants with TSB levels at risk of neurologic damage and with clinical signs and symptoms of ABE. However, available guidelines suggest different thresholds for ET, without differences between haemolytic and non haemolytic jaundice.

**Figure 4:**
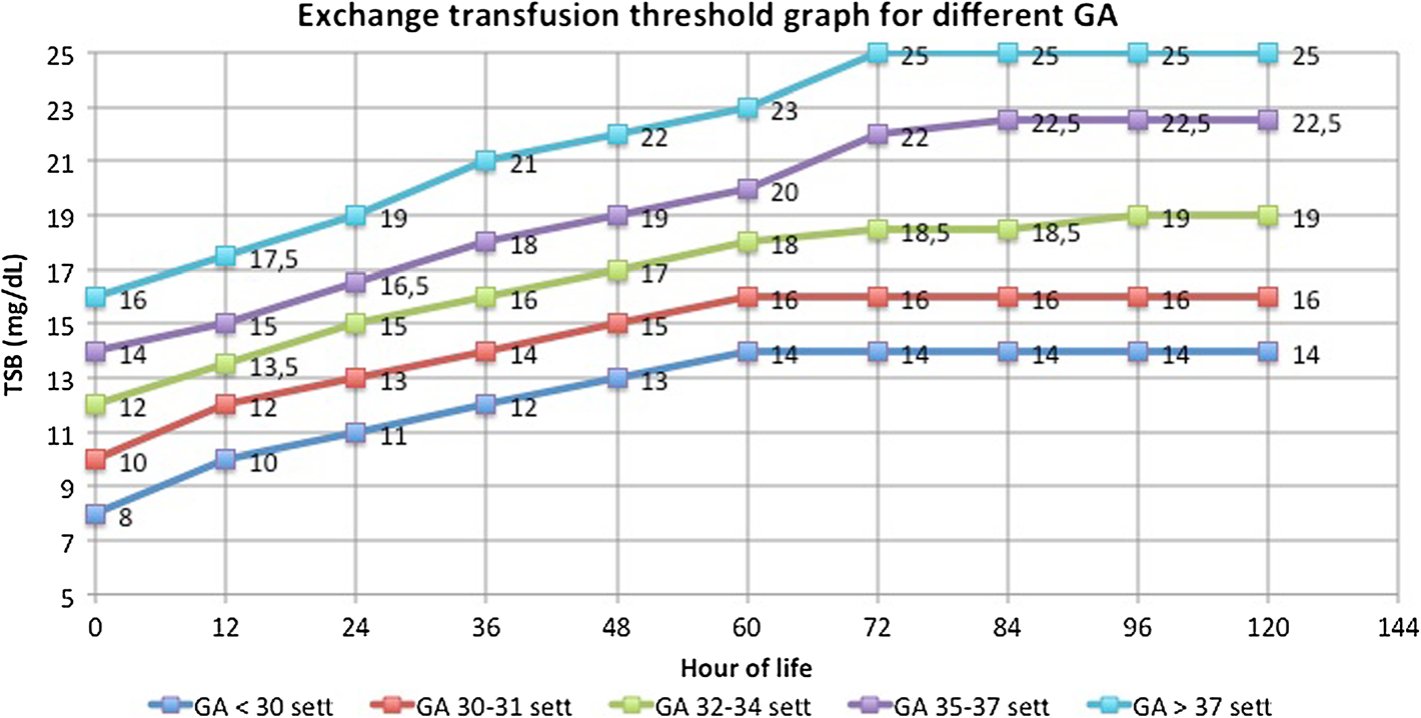
**The graph shows the thresholds for exchange transfusion.** Total bilirubin was plotted against age in hours. The groups of GA were represented with different lines.

TSB threshold for ET in newborns with non haemolytic jaundice are generally 5–6 mg/dL higher than TSB threshold for phototherapy (Figure 4).

**Figure 5:**
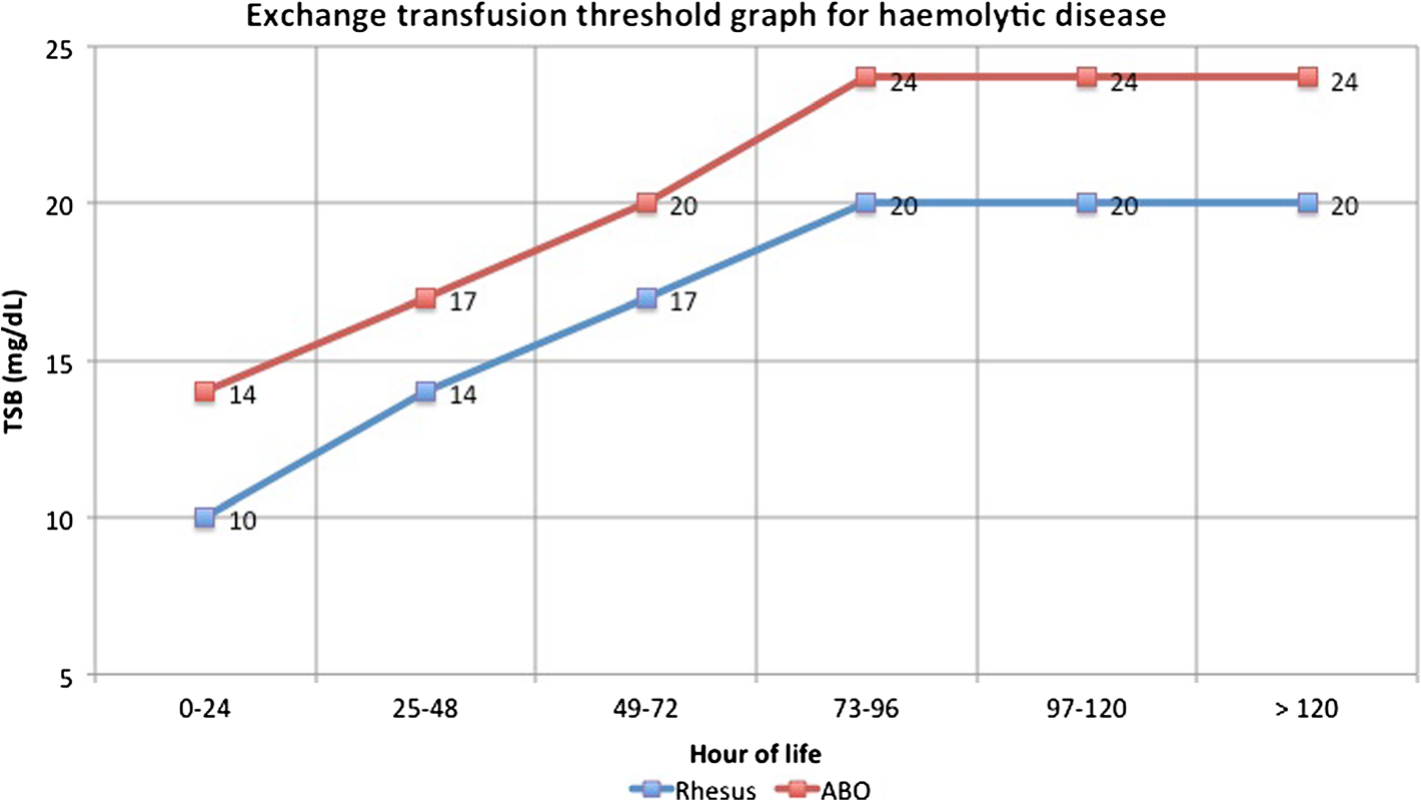
**The graph shows the thresholds for exchange transfusion in case of haemolytic disease.** Total bilirubin was plotted against age in hours.

The TSB threshold for ET in newborns with Rh or ABO haemolytic jaundice is lower than that of newborns with non haemolytic jaundice (Figure 5).

The more conservative approach to the treatment of ABO haemolytic disease compared to Rh haemolytic disease is due to its less aggressive development.

***Recommendations (see Additional file*** ***1*** ***):***

• ET should be performed only by trained personnel in a neonatal intensive care unit with fully monitoring and resuscitation capabilities.

• Heart rate, breath rate, SpO_2_ and body temperature should be monitored during ET and for 12–24 hours after the end of the procedure.

• The most common adverse effects of ET are thrombocytopenia, haemolysis, hypocalcaemia, hypotension, venous thrombosis, hypokalaemia and hypoglycaemia and should be carefully monitored.

• Measurement of TSB should be performed before ET and at the end of the procedure.

• As bilirubin levels may continue to rise after an ET we recommend that TSB is measured every 4–6 hours, and that phototherapy is continued.

• It is recommended to discontinue enteral feeding during ET and for 6 hours from the end of the procedure (evidence level 5).

• Infants underwent ET should be followed up for the development of anaemia after discharge.

### Other therapies

Intravenous immunoglobulins (IVIG) act by preventing haemolysis. IVIG contain pooled IgG immunoglobulins extracted from the plasma of over 1000 blood donors. IVIG at a dose of either 500 mg/kg or 1 gr/kg over 2–4 hours reduce TSB in newborn infants with immune haemolytic jaundice [42,43] and reduce the need for ET (evidence level 1a). IVIG should be administered in the first hours of life to infants with Rhesus haemolytic disease or ABO haemolytic disease when the serum bilirubin continues to rise by more than 0.5 mg/dL per hour.

### Italian registry of kernicterus and hyperbilirubinaemia

The Italian Society of Neonatology instituted the Italian Registry of Kernicterus and Hyperbilirubinaemia (RIKI: Registro Italiano del Kenicterus e dell’Iperbilirubinemia) for monitoring the incidence of kernicterus and severe hyperbilirubinaemia (TSB > 20 mg/dL or need for ET) in Italy. Each new case can be reported through the website of the Italian Society of Neonatology (http://www.neonatologia.it). The objective of this registry is to provide evidences of the guidelines effectiveness through the evaluation of severe hyperbilirubinaemia and kernicterus rate.

## Conclusion

The development of national guidelines provide standardisation of the management of neonatal jaundice. Severe hyperbilirubinaemia in both healthy term or preterm newborn infants continues to carry the potential for complications from ABE. The careful assessment of involved risk factors and the systematic approach to the management of hyperbilirubinaemia are essential to decrease the occurrence of kernicterus.

## Abbreviations

ABE: Acute bilirubin encephalopathy; B/A: Bilirubin/albumin ratio; ET: Exchange transfusion; GA: Gestational age; IVIG: Intravenous immunoglobulin; RBCs: Red blood cells; TSB: Total serum bilirubin; TcB: Transcutaneous bilirubin.

## Competing interests

The authors declare that they have no competing interests.

## Authors’ contributions

CR, CD e FR conceived this study as Task Force for hyperbilirubinaemia of Italian Society of Neonatology. EZ, SP and LC revised literature and collected data from Italian studies. CR, GB and EZ conducted the study on transcutaneous bilirubin and serum bilirubin as predictors of severe hyperbilirubinaemia. GB and CR prepared the draft of the paper. CD, FR and SP made critical revision of the article. All authors read and approved the final manuscript.

## Supplementary Material

Additional file 1 Exchange transfusion procedure. Click here for file
