# Supplementary material for: Italian guidelines for management and treatment of hyperbilirubinaemia of newborn infants ≥ 35 weeks’ gestational age
Source: Ital J Pediatr. 2014 Jan 31;40:11. doi: 10.1186/1824-7288-40-11 (PMC4015911; doi:10.1186/1824-7288-40-11)
Supplement: Additional file 1 — Exchange transfusion procedure. [file 1824-7288-40-11-S1.docx]

**ADDITIONAL FILE 1: EXCHANGE TRANSFUSION PROCEDURE**

**AIMS**

1. Reduction of hyperbilirubinaemia
2. Removal of antibody-coated red blood cells which are source of “potential” bilirubin
3. Correction of the anaemia, if present, and removal of maternal antibody.

**INDICATIONS**

1. Severe anaemia (Hb 6-7 gr/dl; Ht 15-20%) with risk of cardiac failure in newborn infants with haemolytic disease. This condition should be treated with a partial exchange transfusion using packed red blood cells (RBCs) (100 ml).
2. Severe hyperbilirubinaemia without severe anaemia:
   1. Actual hyperbilirubinaemia: the bilirubin level is at risk of neurological damage.
   2. Severe hyperbilirubinaemia: the total serum bilirubin (TSB) continues to rise by more than

0.5 mg/dl per hour because of haemolytic jaundice despite intensive phototherapy

**HAEMODERIVATES**

Reconstituted blood (packed red cells plus fresh-frozen plasma at 3:1 ratio) with 50-60% haematocrit (Htc) should be used.

It is advisable to use :

1. haemoderivates less than 5 days old to reduce risk of hyperkalaemia.
2. washed red blood cells to reduce antibodies titer and the risk of hyperkalaemia.
3. Irradiated blood within 24 hours from irradiation.

**BLOOD TYPING AND CROSS-MATCHING**

Blood must be cross-matched with both the mother and infant blood.

1. In infants with Rh incompatibility: the RBCs must be type Rh-negative.
2. In infants with ABO incompatibility: the RBCs must be type 0, Rh-compatible.
3. RBCs must be used in combination with compatible plasma (for example, RBCs type 0 with plasma type AB)

**CONCOMITANT PROCEDURES**

1. Begin and continue intensive phototherapy
2. Evaluate the administration of IVIG (intravenous immunoglobulins)
3. Perform hematologic and chemistry studies: complete cell blood count, direct antiglobulin test, blood urea nitrogen, electrolytes, liver function panel tests, pH, PaCO_2_, acid-base status and serum glucose.
4. Perform mother’s and infant’s blood typing and cross-matching.

**PERFORM ET IF:**

1. Bilirubin level is ≥ 5 mg/dL (85µmol/L) above ET threshold
2. Bilirubin level exceeds ET threshold after 4 hours of intensive phototherapy
3. Bilirubin continues to rise by more than 1 mg/dL per hour during intensive phototherapy
4. Clinical signs of ABE are present

**PROCEDURE:**

1. Obtain informed consent.
2. Perform ET in an intensive care setting.
3. Use double-volume exchange transfusion with reconstituted blood for treating hyperbilirubinaemia. The normal blood volume in a full term newborn infant is 85 ml/kg. When calculating exchange volumes consider that blood volume in preterm babies may be up to 95 ml/kg.
4. Partial ET should be used in case of severe anaemia (Hb 6-7 gr/dl; Ht 15-20%) at risk of cardiac failure in newborn. In this case use packed RBCs (Ht 75-80%) and exchange 100 ml.
5. Warm the blood up to room temperature.
6. Perform umbilical vein catheterization. The catheter tip placement is in inferior vena cava just above the diaphragm, confirmed by a chest radiograph. If an isovolumetric exchange is to be performed, then an umbilical artery catheter must also be placed and confirmed by radiograph. Otherwise isovolumetric ET can be performed by placing both peripheral venous and arterial access.
7. During the ET heart and respiratory rate, ECG, oxygen saturation, body temperature, and blood pressure should be monitored closely.
8. The push-pull method with a single syringe and a special four-way stopcock assembly permits a single operator to complete the procedure. The actual exchange should be performed slowly in aliquots of 5 to 10 mL, with each withdrawal-infusion cycle approximating 2-3 minutes’ duration. The entire procedure should last 90-120 minutes.
9. Isovolumetric double-volume ET is performed using a double setup, with infusion via the umbilical vein and the withdrawal via the umbilical artery. This method is preferred when volume shifts during simple ET might cause or worsen myocardial insufficiency (for example in babies with hydrops fetalis). Two operators are usually needed: one to perform the infusion and the other to handle the withdrawal.
10. During the procedure take note of the amount and duration of each exchange.
11. The blood should be agitated regularly to maintain a constant Ht.
12. Discontinue enteral feeding during ET and for 6 hours from the end of the procedure.
13. Supplemental calcium gluconate should not be routinely administered. Use 1-2 ml of 10% calcium gluconate if the electrocardiographic abnormalities become evident.
14. Halfway trough the procedure check complete cell blood count, pH, PaCO_2_, acid-base status, bilirubin, electrolytes, and serum glucose.

**AT THE END OF ET**

1. Continue intensive phototherapy and check bilirubin level 2 hour after the end of the ET
2. Perform haematologic and chemistry studies: complete cell blood count, blood urea nitrogen, electrolytes, liver function panel tests, pH, PaCO2, acid-base status, serum glucose and coagulation tests.
3. Monitor in intensive care setting infant’s vital signs for at least 4 hours.

**COMPLICATIONS**

Adverse effects of ET include cardiovascular, haematologic, gastrointestinal, biochemical, and infectious hazards, among others.

1. Infections (for example bacteriaemia or transfusion-transmitted viruses such as CMV, HIV, HCV and HBV).
2. Vascular complications: clot or air embolism, thrombosis.
3. Haemolysis with rebound of bilirubin level.
4. Hypothermia.
5. Coagulopathies that may result from thrombocytopenia or diminished coagulation factors.
6. Hypoglycaemia.
7. Electrolyte abnormalities such as hypocalcaemia, hyperkalaemia, hypomagnesaemia.
8. Necrotizing enterocolitis and death.
